# Spirochaetes Occurring in the Urine of Cases of “Pyrexia of Unknown Origin” (Preliminary Note)

**Published:** 1917-11

**Authors:** 


### BACTERIOLOGICAL AND PATHOLOGICAL

***Spirochaetes Occurring in the Urine of Cases of " Pyrexia of Unknown Origin*** " (Preliminary Note). By S. W. PATTERSON, Major R. A. M. C. (T. C.). Beit Memorial Research Fellow. Abstrated from the *British Medical Journal*, Sept. 29, 1917.

P. for several months has been examining cases of P. U. O. (pyrexia of unknown origin) for spirochaetes in the urine. He believes that he may in this manner determine the character of at least two types of P. U. O. He lists the cases in which he has found spirochaetes as follows :

| | |
|---|---|
| Trench nephritis. . . . . . . . . . . . . . . . . | 3 cases. |
| Pyelonephritis, with abcesses of the lungs. . . . | 1 case. |
| Relapsing P.U.O.. . . . . . . . . . . . . . . . . | 15 cases. |
| Myalgia following P.U.O. . . . . . . . . . . . . | 1 cases. |
| N. Y. D. (not yet diagnosed) appendicitis. . . . | 5 cases. |

The author describes the spirochaetes as being in length one and one half the diameter of a red blood corpuscle, thin, with ends tapering. They have from five to eight more or less regular turns, and may lie straight or bowed. The spirals are coarser than in the *pallida* and *Obermeier* varieties. They resist staining except by Indian ink methods. They are found more abundantly in the urine during or immediately after a rise in temperature, and they may be so scarce in the intervals of low temperature as to be practically absent.

The two types of P. U. O. which especially engage the author's attention, he describes as follows :

" 1. A disease with acute onset with chills and vomiting, pain in the abdomen, usually more marked in the right upper quadrant, with continued fever for several days, often with enlargement of the spleen and herpes of the lips. The pulse rate is not increased and is usually slowed to 50 or 60 beats a minute in convalescence. There is leucocytosis of 12 000 to 26 000 with relative increase in the large mononuclears. The cases, if an attempt is made at diagnosis, come to the base as " appendicitis? ", or " N. Y. D. abdominal. " I recognize that these are cases which presented nothing but abnormal temperature, malaise, and slow pulse. " The author also remarks that although Stokes had previously noted that guinea pigs into which the blood of patients with these symptoms had been injected died of spirochaetosis, that author had not found spirochaetes in the urine. P. attributes his success in this particular to the improved technique employed.

" 2. The relapsing type of P. U. O. (trench fever) with the characteristic periodic rises of temperature, myalgic and periosteal pains, and leucocytosis with or without enlargement of the spleen ".

The author's method in the urine test consisted in carefully preventing contamination of the sample. The urine was centrifuged, the supernatant fluid poured off, the deposit washed with sterile water, and recentrifuged. The spirochaetes appear to be of low specific gravity and require at least 10 minutes of centrifuging. The lower layer of supernatant urine should not be removed before recentrifuging. The author thus found it easier to discover the organism among some debris, than to find isolated specimens on a comparatively clean slide. The following methods of dealing with the deposit were adopted because dark-ground illumination was not available ; Indian Ink (" chin-chin " liquid pearl ink Watson); Tannic Acid and Carbol Fuchsin (Renaux and A. Wilmaers), and Tannic Acid and Silver Nitrate (Fontana). The organisms resisted staining by the Leishman, Romanowsky, or Giemsa's stains and required a mordant method. The Indian Ink method was successful.

